# Efficacy and safety of 3-month dual antiplatelet therapy in patients after mechanical thrombectomy for acute ischemic stroke: a retrospective study

**DOI:** 10.3389/fneur.2024.1374093

**Published:** 2024-04-15

**Authors:** Liang Li, Zhihui Liang, Cong Lin, Bao Cui, Qiong Jia

**Affiliations:** Department of Intervention, Bethune International Peace Hospital, Shijiazhuang, China

**Keywords:** acute ischemic stroke, mechanical thrombectomy, dual antiplatelet drug therapy, efficacy, safety

## Abstract

**Background:**

Mechanical thrombectomy (MT) is one of the effective treatment methods for acute ischemic stroke (AIS), which requires a period of dual antiplatelet therapy (DAPT) after endovascular treatment. This study aimed to compare the efficacy and safety of 3-month DAPT and 1-month DAPT in AIS patients receiving MT through a retrospective study.

**Methods:**

AIS patients who received MT from May 2018 to March 2023 were grouped into a 1-month group (1-M group) and a 3-month group (3-M group) according to the duration of DAPT after MT. The primary outcome was the mRS score at 90 days. Secondary outcomes included a good prognosis (mRS score of 0–2) at 90 days post-surgery, 6-month mortality, recurrence of cerebral infarction, Barthel's index, Montreal Cognitive Assessment (MoCA) score, and incidence of symptomatic intracranial hemorrhage (sICH) during hospitalization.

**Result:**

A total of 147 patients with AIS were included in the final analysis, with 78 patients in the 1-M group and 69 patients in the 3-M group. The baseline and neurological characteristics were comparable between both groups. At 3-month follow-up, a total of 61 patients had an mRS of 0–2 at 90 days, with an average mRS of 3.3 ± 0.9 for all patients. There was no statistically significant difference in the mRS between the two groups of patients at 90 days (P > 0.05). There was no statistically significant difference in the mortality rate and incidence of sICH between the two groups of patients during the 6-month follow-up period (P > 0.05), but the recurrence rate of AIS in the 3-M group was lower than that in the 1-M group (*P* < 0.05). The improvement of Barthel index and MoCA in patients in the 3-M group was higher than those in the 1-M group at 6 months but not statistically different (*P* > 0.05).

**Conclusion:**

For AIS patients undergoing mechanical thrombectomy, compared to 1-month DAPT, 3-month DAPT can reduce the recurrence rate of IS during a 6-month follow-up period, without increasing the mortality and risk of cerebral hemorrhage.

## Introduction

Stroke is one of the major diseases with a global burden, with acute ischemic stroke (AIS) being the predominant (1). If ischemic stroke is not treated with timely and effective vascular recanalization, it can lead to neurological damage, decreased quality of life, and even death (2) in patients. Intravascular intervention is one of the main treatment methods for acute ischemic stroke, which can significantly improve the prognosis of AIS patients within 6 h of onset (3). In 2015, five randomized controlled trials showed that mechanical thrombectomy (MT) within 6 h of onset was more effective in improving 90-day neurological function in patients with acute anterior circulation large vessel occlusive stroke (ACLVOS) when compared to drug treatment (4–8). In recent years, some clinical studies have observed the efficacy and safety of MT on AIS patients within 24 h of onset. The results of the 2018 DAWN and DEFUSE 3 studies indicate that MT within 6–24 h of AIS onset can still benefit patients (9, 10). AIS can be classified into atherosclerosis, cardiogenic thromboembolism, and other types based on the mechanism (TOAST classification), among which arterial atherosclerosis is the predominant type. The main pathophysiological mechanism of arterial atherosclerosis is the instability of cerebral artery atherosclerotic plaque, which can form a thrombus to block the blood vessels when the plaque ruptures. Therefore, MT requires the use of dual antiplatelet drugs for therapy. In some guidelines for AIS patients, the use of a combination of aspirin with clopidogrel is recommended for at least 1 month after mechanical thrombectomy (11, 12). However, the pathological basis of atherosclerotic AIS is similar to that of acute coronary syndrome (ACS). After interventional treatment, ACS often requires 1–2 years of dual antiplatelet therapy (DAPT). Considering that prolonged DAPT for AIS after MT may increase the risk of cerebral hemorrhage, the current duration of DAPT is relatively short. In clinical practice, we used DAPT on some AIS patients for 3 months. This study aimed to investigate the efficacy and safety of 3-month DAPT in AIS patients at 3 months after MT through a retrospective study.

## Methods

### Study population

This study included AIS patients who were hospitalized from May 2018 to March 2023 for retrospective analysis. According to the duration of DAPT after MT, the patients were classified into a 1-month group (1-M group) and a 3-month group (3-M group). The inclusion criteria were as follows: age is ≥ 18 years; the diagnostic criteria for AIS is met (13); the time from onset to admission is ≤ 24 h; and the CT scan at admission showed significant ischemic penumbra and met the imaging screening criteria in the DEFUSE 3 study (10): core infarct volume of <70 mL, ischemic volume/core infarct volume of ≥ 1.8 mL, mismatch volume of ≥ 15 mL; at admission, the National Institute of Health Stroke Scale (NIHSS) score was ≥ 6 points; the patient received regular follow-up after discharge; and the clinical data are complete. The exclusion criteria were as follows: concomitant malignant tumors, renal failure, and heart failure; rheumatic diseases; received coronary artery stent implantation within 1 year prior to the onset of stroke; atrial fibrillation and cardiogenic cerebral embolism; at admission, CT examination showed concurrent cerebral hemorrhage and an Alberta stroke program early CT score (ASPECTS) of <6; loss of follow-up. This study was approved by the Ethics Committee of Bethune International Peace Hospital (approval number: 2021-KY-142), and written informed consent was waived.

### Mechanical thrombectomy

After comprehensive evaluation on admission, the patient was immediately treated with MT. After sufficient local infiltration anesthesia with lidocaine, the Seldinger technique was used to puncture the right femoral artery, and then, an 8F arterial sheath was inserted, followed by aortic arch angiogram and cerebral angiography. After identifying the location of the thrombus, a thrombectomy was conducted. Stent thrombectomy: After passing through the occluded area and confirming the true lumen of the blood vessel, the microwire and microcatheter were inserted into the Solitaire FR stent (Medtronic Ltd, USA), and then, the thrombectomy device was retrieved under negative pressure. Thrombolysis: under the guidance of microwires, the Sofia aspiration catheter (MicroVention Ltd, USA) or ACE aspiration catheter (Penumbra Inc., USA) was inserted until it met the thrombus, and then, negative pressure was maintained and aspiration was performed for 90 s. After removing the thrombus, a repeated cerebral angiography was performed to evaluate the treatment effect and the presence of bleeding. If the residual stenosis rate in the local area was >70% or if a small thrombus escaped from the distal end, measures such as balloon angioplasty, stent implantation, and intra-arterial thrombolysis were used for remediation.

### Postoperative medication

Patients in the 1-M group received 100 mg oral aspirin and 75 mg clopidogrel daily for 1 month after endovascular treatment. The 3-M group received DAPT treatment according to the same protocol, lasting for 3 months. After the completion of DAPT, aspirin was continued for at least 1 year. If patients have diabetes, hypertension, and coronary heart disease, they should be treated according to relevant guidelines. Specifically, both groups of patients received atorvastatin at a daily dose of 20–40 mg.

### Follow-up

In this study, all patients underwent regular outpatient follow-up at 1 month, 3 months, and 6 months after surgery. The information of follow-up included the following: postoperative mRS score at 90 days and adverse events, mainly including death, recurrence of cerebral infarction, and symptomatic intracranial hemorrhage (sICH). The follow-up was conducted in an outpatient clinic, by telephone, or through WeChat.

### Data collection and outcomes

All data were collected from the electronic medical record system. Baseline data include age, gender, smoking history, alcohol use, disease history, physical examination results, laboratory test results, and imaging results on admission. Information on in-hospital management and all follow-up outcomes was collected. The primary outcome was the mRS score at 90 days. The mRS score was assessed by physical examination in an outpatient clinic 90 days after onset. Secondary outcomes included a good prognosis (mRS score of 0–2) 90 days after the endovascular intervention, mortality, recurrence of cerebral infarction, Barthel's index, Montreal Cognitive Assessment (MoCA) score, and incidence of sICH at the 6-month follow-up. The prespecified adverse event was sICH defined by the National Institute of Neurological Disorders and Stroke (NINDS) criteria.

### Statistical analysis

Statistical Package for the Social Sciences (SPSS, version 24.0, USA) was used to analyze data. Continuous data with normal distribution were presented as mean ± standard deviation and compared between two groups using a two-sample independent *t* test. Category data were presented as numbers (percentages) and compared between two groups using the chi-square or Fisher's exact test. A two-sided *p* < 0.05 indicates a statistical difference.

## Results

### Baseline characteristics

According to the inclusion and exclusion criteria, a total of 147 patients with AIS were included in the final analysis. Among these patients, there were 108 (73.5%) patients with hypertension, 44 (29.9%) with type 2 diabetes, and 99 (67.3%) with hyperlipidemia. Seventy-eight patients received 1-month DAPT with 100 mg aspirin daily and 75 mg clopidogrel daily, and the remaining 69 patients were treated with DAPT for 3 months. A comparison between the 1-M group and the 3-M group showed no statistical difference regarding baseline characteristics (*P* > 0.05, Table 1).

**Table 1:** Baseline characteristics.

**Characteristics**	**1-M group (*n =* 78)**	**3-M group (*n =* 69)**	**t / X^2^ value**	***P***
Age (years)	63.1 ± 7.8	64.3 ± 9.1	0.861	0.391
Male (*n*, %)	54 (69.2)	51 (73.9)	0.393	0.531
Smoking (*n*, %)	24 (30.8)	28 (40.6)	1,541	0.214
Alcohol use (*n*, %)	41 (52.6)	30 (43.5)	1.210	0.271
Hypertension (*n*, %)	55 (70.5)	53 (76.8)	0.745	0.388
Diabetes (*n*, %)	21 (26.9)	23 (33.3)	0.717	0.397
Hyperlipidemia (*n*, %)	52 (66.7)	47 (68.1)	0.035	0.852
BMI (kg/m^2^)	25.3 ± 3.9	24.8 ± 4.0	0.766	0.445
SBP (mmHg)	154.6 ± 13.2	157.1 ± 14.9	1.079	0.282
FG (mmol/L)	7.3 ± 1.1	7.0 ± 1.2	1.581	0.116
TC (mmol/L)	5.9 ± 0.8	5.8 ± 1.0	0.673	0.502
TG (mmol/L)	2.8 ± 0.9	3.0 ± 0.8	1.416	0.159
PT (s)	12.5 ± 1.9	12.7 ± 2.0	0.621	0.535
APTT (s)	30.3 ± 4.5	29.6 ± 4.8	0.912	0.363

BMI, body mass index; SBP, systolic blood pressure; FG, fasting glucose; TC, total cholesterol; TG, triglyceride; PT, prothrombin time; APTT, activated partial thrombin time.

### Neurological characteristics

The time from onset to initiation of MT in patients is 3–14 h, with an average of 6.1 ± 1.5 h. A comparison of neurological characteristics between the 1-M group and the 3-M group also found no statistically significant difference (all *P* > 0.05, Table 2).

**Table 2:** Neurological characteristics.

**Characteristics**	**1-M group (*n =* 78)**	**3-M group (*n =* 69)**	**t / X^2^ value**	***P*-value**
Onset to MT time (h)	5.9 ± 1.4	6.3 ± 1.7	1.564	0.120
NIHSS score	17.6 ± 3.1	18.2 ± 3.5	1.102	0.272
Diameter of lesions (mm)	3.5 ± 0.8	3.7 ± 0.9	1.426	0.156
Culprit vessels (*n*, %)			0.237	0.626
MCA	49 (62.8)	46 (66.7)		
ICA	29 (37.2)	23 (33.3)		
IDS (*n*, %)	4 (5.1)	3 (4.3)	0.028	0.868

MTG, mechanical thrombectomy group; CG, control group; MoCA, Montreal cognition assessment; TOAST, Trial of Org 10172 in Acute Stroke Treatment; IDS, intracranial decompression surgery.

### Follow-up outcomes

The follow-up results (Table 3) showed that there were a total of 61 patients in both groups with a 90-day mRS of 0–2, and the average mRS of all patients at 90 days was 3.3 ± 0.9. There was no statistically significant difference in the mRS between the two groups of patients 90 days after surgery (P > 0.05). There was no statistically significant difference in the mortality rate and incidence of sICH between the two groups of patients during the 6-month follow-up period (P > 0.05), but the AIS recurrence rate in the 3-M group was lower than that in the 1-M group (*P* < 0.05). Three cases of sICH occurred in patients with poor blood pressure control, and the sICH was located at the internal capsule artery. We also observed that patients in the 3-M group had higher improvement in Barthel scores and MoCA improvement at 6 months compared to the 1-M group but did not show statistically significant differences (*P* > 0.05).

**Table 3:** Comparison of follow-up results between the two groups of patients.

**Characteristics**	**1-M group (*n =* 78)**	**3-M group (*n =* 69)**	**t / X^2^ value**	**P**
**3-month follow-up**				
90-d mRS	3.4 ± 0.9	3.2 ± 1.0	1.276	0.204
mRS 0-2 (*n*, %)	31 (39.7)	30 (43.5)	0.210	0.646
**6-month follow-up**				
Death (*n*, %)	5 (6.4)	6 (8.7)	0.276	0.599
AIS recurrence (*n*, %)	9 (11.5)	2 (2.9)	3.948	0.047
sICH (*n*, %)	1 (1.3)	2 (2.9)	0.011	0.914
Barthel improvement	37.6 ± 5.5	39.3 ± 5.7	1.839	0.068
MoCA improvement	8.9 ± 2.6	9.7 ± 2.9	1.764	0.080

sICH, symptomatic intracranial hemorrhage; MoCA, Montreal cognition assessment.

## Discussion

The preliminary results of this study indicate that 3-month DAPT after MT can significantly reduce the risk of AIS recurrence in AIS patients while not increasing the risk of death and sICH. Our study also found that 3-month DAPT showed a trend toward further improvement in patients' daily living conditions and cognitive abilities.

For AIS patients with non-cardiogenic embolism, the mechanism of culprit vessel occlusion is similar to that of acute myocardial infarction, which is the formation of a thrombus caused by the rupture of an unstable atherosclerotic plaque, leading to vascular occlusion. These AIS patients are often known to have hypertension, diabetes, and hyperlipidemia. In this study, patients with hypertension, diabetes, and hyperlipidemia accounted for 73.5%, 29.9%, and 67.3%, respectively. Moreover, this study excluded AIS caused by cardiogenic thrombus, so the pathogenesis of AIS patients in this study may be atherosclerosis. In the treatment of unstable atherosclerotic plaque, antiplatelet drugs are mainly used to prevent thrombosis, while intensive statin therapy is used to stabilize plaque (14, 15). In this study, patients received long-term treatment with aspirin (100 mg daily) and atorvastatin. The difference was that patients in the 3-M group received clopidogrel for 3 months after surgery, while patients in the 1-M group only used it for 1 month.

Antiplatelet therapy is one of the main treatment measures for patients with acute ischemic stroke. In the CHANCE study, Wang et al. compared the efficacy and safety of DAPT with those of aspirin alone in the treatment of acute minor stroke or transient ischemic attack in a randomized controlled trial. The results showed that, compared with aspirin alone, DAPT significantly reduced the risk of stroke within 90 days after the attack, while it did not increase the risk of cerebral hemorrhage (16). Since Wang et al.'s (16) study, the era of DAPT treatment for ischemic stroke began. At present, for patients with ischemic stroke who receive endovascular treatment and DAPT treatment, existing guidelines recommend that patients with extracranial arch artery lesions receive DAPT for at least 30 days after surgery, and then, they were switched to aspirin alone. For patients with intracranial arterial lesions, it is recommended to switch to aspirin alone after 3–6 months of DAPT (17, 18). If a stent is implanted, the recommended duration of DAPT is 1–3 months, followed by long-term aspirin treatment (19). There is currently insufficient evidence to support clear recommendations from guidelines or consensus on the duration of DAPT use for AIS patients undergoing MT after surgery.

The antiplatelet mechanisms of aspirin and clopidogrel are different, and the combination of the two drugs can have a stronger effect than when using a single drug. Administering DAPT during the acute phase of AIS can effectively prevent thrombus formation at the target lesion site after MT. After a certain period of comprehensive treatment, including antihypertensives, hypoglycemics, and statin drugs, the blood vessels at the target lesion site can be repaired and endothelial function can be restored. However, the exact duration required for endothelial function recovery is currently unclear. Previous studies have focused more on the use of antiplatelet drugs in patients with cerebrovascular disease after stent placement. In the Stenting and Aggressive Medical Management for Preventing Recurrent Stroke in Intracranial Stenosis (SAMMPRIS) study, patients received DAPT with 75 mg/day of clopidogrel and 325 mg/day of aspirin for 3 months. At an average follow-up of 11.9 months, safety evaluations indicated that the incidence of sICH in the stent implantation group compared to that in the intensified drug group was 3.6% (8 cases) vs. 0.4% (1 case) (20–22). Hassan et al. (23), in a previous study, observed the incidence of intracranial and systemic bleeding events, recurrent ischemic stroke, and death in patients treated with dual antiplatelet therapy for more than 1 month. In the study, DAPT was administrated in 110 patients following endovascular treatment with a median duration of 3 months. During the study period, there were two bleeding events (one intracranial and one gastrointestinal), one ischemic stroke, and no deaths (23). It should be noted that, in some studies in Europe and America, the dosage of aspirin is significantly higher than that in Chinese patients. In clinical practice in China, except for the load dose of 300 mg given during acute AIS intervention surgery, the long-term dose after endovascular intervention is 100 mg per day (12). Therefore, when Chinese patients use DAPT, the daily dosage of aspirin is significantly lower than that in European and American patients, so the risk of cerebral hemorrhage may be lower than that in European and American patients. Three patients in this study developed sICH during a 6-month follow-up period, which may be related to uncontrolled hypertension.

Our research findings also suggest that, compared to 1-month DAPT, 3-month DAPT has a trend toward further improvement in patients' daily living and cognitive abilities, which was suggested by slight improvement in Barthel index and MoCA scores. The relatively small sample size of the present study and short follow-up duration might be the reasons for no statistical significance. This finding may be related to the more effective preventive effect of longer DAPT on ischemic stroke, especially in the prevention of minor ischemic stroke or transient ischemic attack. However, currently, there is a lack of evidence to support it.

There are some limitations in this study. First, this study is a single-center study with a small sample size. Second, this study is a retrospective study, and some patients were excluded due to incomplete data, which may lead to case selection bias. Third, there is insufficient information on the retrospective data to assess the presence of asymptomatic mild cerebral hemorrhage. In the future, multicenter, large-sample randomized controlled studies with long-term follow-up are needed to further evaluate the efficacy and safety of 3-month DAPT in AIS patients undergoing mechanical thrombectomy.

## Conclusion

The results of this study indicate that a 3-month DAPT can safely and effectively reduce the risk of IS in AIS patients undergoing mechanical thrombectomy during a 6-month follow-up period.

## Data availability statement

The original contributions presented in the study are included in the article/supplementary material, further inquiries can be directed to the corresponding author.

## Ethics statement

The studies involving humans were approved by the Ethics Committee of Bethune International Peace Hospital. The studies were conducted in accordance with the local legislation and institutional requirements. The Ethics Committee/institutional review board waived the requirement of written informed consent for participation from the participants or the participants' legal guardians/next of kin due to the retrospective nature of the study.

## Author contributions

LL: Conceptualization, Formal analysis, Funding acquisition, Investigation, Methodology, Writing – original draft, Writing – review & editing. ZL: Data curation, Formal analysis, Investigation, Writing – original draft. CL: Data curation, Formal analysis, Investigation, Writing – original draft. BC: Data curation, Resources, Software, Validation, Writing – original draft. QJ: Investigation, Methodology, Writing – original draft.
